# Therapeutic Potential of Marine Probiotics: A Survey on the Anticancer and Antibacterial Effects of *Pseudoalteromonas* spp.

**DOI:** 10.3390/ph16081091

**Published:** 2023-08-01

**Authors:** Osita C. Eze, Dinebari P. Berebon, Stephen C. Emencheta, Somtochukwu A. Evurani, Chibundo N. Okorie, Victor M. Balcão, Marta M. D. C. Vila

**Affiliations:** 1 Department of Pharmaceutical Microbiology and Biotechnology, Faculty of Pharmaceutical Sciences, University of Nigeria, Nsukka 410001, Nigeria; osy.eze@unn.edu.ng (O.C.E.); somtochukwu.evurani@unn.edu.ng (S.A.E.); chibundo.okorie@unn.edu.ng (C.N.O.); 2 PhageLab-Laboratory of Biofilms and Bacteriophages, University of Sorocaba, Sorocaba 18023-000, Brazil; victor.balcao@prof.uniso.br (V.M.B.); marta.vila@prof.uniso.br (M.M.D.C.V.); 3 Department of Biology and CESAM, University of Aveiro, Campus Universitário de Santiago, P-3810-193 Aveiro, Portugal

**Keywords:** marine probiotics, *Pseudoalteromonas* spp., therapeutic effects, anticancer, antibacterial

## Abstract

Due to the increasing limitations and negative impacts of the current options for preventing and managing diseases, including chemotherapeutic drugs and radiation, alternative therapies are needed, especially ones utilizing and maximizing natural products (NPs). NPs abound with diverse bioactive primary and secondary metabolites and compounds with therapeutic properties. Marine probiotics are beneficial microorganisms that inhabit marine environments and can benefit their hosts by improving health, growth, and disease resistance. Several studies have shown they possess potential bioactive and therapeutic actions against diverse disease conditions, thus opening the way for possible exploitation of their benefits through their application. *Pseudoalteromonas* spp. are a widely distributed heterotrophic, flagellated, non-spore-forming, rod-shaped, and gram-negative marine probiotic bacteria species with reported therapeutic capabilities, including anti-cancer and -bacterial effects. This review discusses the basic concepts of marine probiotics and their therapeutic effects. Additionally, a survey of the anticancer and antibacterial effects of *Pseudoalteromonas* spp. is presented. Finally, marine probiotic production, advances, prospects, and future perspectives is presented.

## 1. Introduction

Natural products (NPs) are highly structurally diversified, ubiquitous life forms and derivatives of living organisms and minerals. They have been used extensively, especially in traditional medicine, to manage diverse diseases [1]. They have also contributed immensely to drug discovery in making current conventional drugs, serving as a direct source of medicinal substances, raw materials in drug production, lead compound design prototypes, and taxonomic biomarkers for new drug search and discovery [2]. NPs are associated with prominent, apparent beneficial properties [3] (as shown in Table 1) against conventional drugs or radiotherapy (as implicated in cancer management), including minimal side effects, toxicity, allergenicity, and low-cost isolation, identification, characterization, and production.

Synthetic drugs, including antibiotics used in aquaculture, primarily aim to prevent infection, leading to fatalities of “aquatic products” with correspondingly low productivity. However, their irrational use undermines the purpose. Although used optimally, it does not guarantee a clean bill of zero health risks to humans [4]. These drugs, including chloramphenicol, sulfamethazine, oxytetracycline, and furazolidone, remain residues in aquatic animals, with their bioaccumulation and biomagnification linked to human carcinogenicity [5,6,7]. Cancer is a leading cause of death globally, with yearly increasing cases [8] and an estimated 19 and 10 million new occurrences and deaths, respectively, in 2020 [9]. The risk/predisposing factors impact the DNA repair system following mutation in a single cell [10]. The pioneer single precancer cell produces many neoplastic cells and tissues via specific mechanisms, leading to undesirable physiological states of the cell, the surrounding cells, tissues, and the entire system. Clinically managing or controlling cancer cells often involves using single and multiple chemotherapeutic drugs to kill or destroy benign or malignant tumor cells and applying radiation to target sites [11,12]. Chemotherapy usually consists of administering high doses of synthetic chemicals for extended durations. Thus, the chemotherapeutic management of cancer has obvious negative implications. Most notable are the drop in the quality/standard of living of most recipient patients due to the extensive side effects of chemotherapeutic drugs on the surrounding cells and systems, the cost of acquiring and maintaining the therapy, and the contribution of chemicals to increasing drug resistance [10]. Radiation therapy has negative implications, including bioaccumulation of radiation, inadequate replacement of damaged stem cells, and injury [13].

NPs are also being exploited to produce anti-infective agents, especially with emerging infectious diseases, antibiotic resistance, and the dearth of new chemotherapeutic agents. Furthermore, the importance of NPs with immunomodulatory and antioxidant properties in combating bioterrorism cannot be overemphasized. The inadequacies in current therapeutic options highlight the need for safer, cheaper, and cost-effective alternative therapies, mainly from various natural products (NPs) that are abundant in the environment. One awakening mindset involves using marine probiotics for their inherent enormous potential [3]. Marine probiotics are a group of natural microbial dwellers of the marine ecosystem that, through their biological activities, apparently contribute to improving aquatic life forms through disease resistance, growth, stress tolerance, reproduction, and water quality [14,15]. They are being exploited for possible utilization in disease prevention and management and have many genera and species belonging to different phyla, including *Pseudoalteromonas* spp. The cold-adapted *Pseudoalteromonas* spp. are diverse understudied marine probiotics usually resident in the extreme marine environment and has shown great potential for therapeutic and biotechnological applications [16]. Despite limited literature, they are reported to elicit bioactive metabolites/compounds with potential medicinal applications, including anti-cancer and -bacterial therapies [17,18]. Also, there is no existing review on the aggregation of studies on the species. This review aims to discuss the potential of marine probiotics with a specific focus on *Pseudoalteromonas* spp. for cancer prevention, management, and antibacterial effects. The first is the background, basic concepts, sources, and classifications of marine probiotics; second, some general therapeutic effects of marine probiotics; third, an extensive survey of the anticancer and antibacterial effects of *Pseudoalteromonas* spp; and finally, a general review of marine probiotic production, prospects, advances, and future perspectives.

**Table 1:** Comparison of side effects of chemotherapeutic drugs, radiation (as applied in cancer management), and natural products.

S/N	Therapy	Side Effects	Therapy Cost	Production Cost	Drug Resistance	Bioaccumulation	Mutation	Injury	Reference
1	Chemotherapeutic drugs	Extensive side effects	High cost of acquiring and maintaining therapy	High cost of development and production of candidate drugs	Contribution of chemicals to increasing drug resistance	Bioaccumulation of chemicals	Not applicable	Not applicable	[19]
2	Radiation	Extensive side effects	High cost of acquiring and maintaining therapy	Not applicable	Not Applicable	Bioaccumulation of radiation	Induction of mutation	Inadequate replacement of damaged stem cells	[13]
3	Natural products	Minimal side effects	Relatively low cost of acquiring and maintaining therapy	Relatively low cost of isolation, identification, characterization, and production	Little or no contribution to resistance	Little or no accumulation	No induction of mutation	No injuries	[20]

## 2. Methodology

This paper is organized into three parts. The first part is a general review of the background of marine probiotics, sources, and some therapeutic effects. The second part is an extensive survey presenting relevant literature on the involvement of *Pseudoalteromonas* spp. in the prevention and management of cancer and bacterial diseases. The third section reviews marine probiotic production, prospects, advances, and future perspectives.

For the second part, the classical literature search of titles, abstracts, and keywords across four databases, including PubMed, ScienceDirect, Scopus, and google scholar (as a secondary database), was done using the ‘*Pseudoalteromonas* spp.’, ‘cancer’, ‘anticancer’, and antibacterial’, as keywords. The study used most original articles published in the last ten (10) years (2013–2023). The literature search was performed using the Boolean connectors: “And” or “Or” where necessary. Original articles reporting the anticancer and antibacterial effects of *Pseudoalteromonas* spp. in the stated years were included. All articles outside the study year range, not written in English, not reporting on *Pseudoalteromonas* spp., review articles, and not reporting anticancer and antibacterial effects were excluded. Some of the limitations of the review include the exclusion of subscription-based articles requiring payment for access. Also, most articles obtained using the keywords were review articles.

## 3. Probiotics, Sources, and Classifications

Probiotics resulting from the Greek words “Pro bios,” which translates to “for life” [21], are a class of microorganisms, including bacteria, viruses (such as bacteriophages), and fungi (yeast and mold) that can be ingested or topically applied for dietary and numerous medicinal (physiological and immunological) purposes [8,22,23]. Examples include *Bifidobacterium*, *Streptococcus*, *Bacillus*, *Escherichia coli*, *Lactobacillus*, *Saccharomyces*, *Coccobacilli*, and *Propionibacterium* and are in varying classifications, mechanisms of action, and corresponding functions. They preserve specific qualities, including, but not limited to, the ability to inhibit pathogens in the gut, navigate and survive via intestinal transit and gastric/bile secretions, adhere to the mucosa of the intestine, have immunomodulating and other biological effects [10,24]. Probiotic strains should be thoroughly characterized, safe for the intended application, backed by at least one successful human clinical trial per generally accepted scientific criteria, and alive in adequate numbers in the product at the time of usage [25]. Other considerations in choosing for medicinal purposes include non-toxicity, non-pathogenicity, beneficial effects, and appreciable shelf life. Probiotics have been applied in managing diverse medical conditions [10]. Also, several preclinical and clinical trials have suggested potent therapeutic applications.

Probiotics are traditionally used and present in fermented food products (e.g., milk, yogurt, cheese, buttermilk, kombucha, sauerkraut, and tempeh) and supplements [10]. The major classifications of probiotics include lactic acid (e.g., *Streptococcus*, *Lactobacillus*, *Enterococcus*, and *Bifidobacterium*) and non-lactic acid strains (e.g., *Bacillus*, *Clostridium*, and *Propionibacterium*), yeasts (e.g., *Saccharomyces*, *Candida*, *and Debaryomyces*) and viruses, with each group exhibiting different mode of operation [26]. Based on ecosystems, terrestrial and aquatic or marine-based probiotics are the classifications [27]. Probiotics are involved in the production of inhibitory substances which prevent the adhesion of pathogens to the intestinal epithelium, direct inhibition of gram-negative bacteria, regulation of short-chain fatty acids, downregulation of proinflammatory cytokines, colonization of intestinal permeability, regulation of electrolyte adsorption, maintenance of the immune response of the intestine, and maintenance of lipid metabolism [26].

## 4. Marine Probiotics and General Therapeutic Potentials

Marine probiotics abound in the aquatic ecosystems. Indigenous and exogenous microbiota from aquatic animals is the primary source for isolating probiotic strains, with the *Lactobacilli*, *Pseudomonas*, *Shewanella*, *Fluorescens*, *Phaeobacter*, and *Bifidobacteria* being the most common genera [28,29]. They have many beneficial roles, including the protection of aquatic life forms. They naturally act as disease control agents in aquatic plants and fishes, promote growth, improve digestion and immune systems, provide sources of nutrients, improve water quality, encourage reproduction, form beneficial relationships with the host and the environment, enhance gut health and immune response in higher animals, and improve human health by preventing and treating various diseases such as cancer, gastrointestinal disorders, respiratory infections, and skin diseases [21,30,31]. They also engage in the blockage of the pathogen’s ability to utilize certain nutrients, prevention of their attachment to the host environment, distortion of the enzymatic activities of the pathogens, enhancement of the quality of water, stimulation of the immune system, and improvement of host nutrition. Marine probiotics are a promising source of novel bioactive compounds with anticancer, antibacterial, immunomodulatory, antioxidant, anti-inflammatory, and antiviral properties, as have been implicated in several studies [27,32,33,34,35,36,37,38,39].

Studies have detailed the potential of yeast as a probiotic for cancer management [40], especially the isolates from marine ecosystems [41,42,43,44,45] and floras of the gut system [42]. Various marine yeast microbiota genera with potential anticancer effects have been identified, including *Saccharomyces*, the most studied genera, particularly in colorectal cancer, as discussed by Sambrani et al. [40]. Others are *Candida*, *Debaryomyces*, *Kluyveromyces*, *Pichia*, *Saccharomyces*, *Cryptococcus*, *Rhodosporidium*, *Rhodotorula*, *Sporobolomyces*, *Mrakiafrigida*, *Guehomyces pullulans*, *Rhodotorula*, *Rhodosporidium*, *Yarrowialipolytica*, *Aureobasidium*, *Metschnikowia* spp., *Torulopsis* spp., *Pichia*, *Kluyveromyces*, *Williopsis*, *Pseudozyma* spp., *Hansenula*, *Trichosporon*, *Filobasidium*, *Leucosporidium*, *Mrakiafrigida*, *Guehomyces pullulans*, *Metschnikowia*, *Rhodotorula*, *Cystobasidium*, and *Yamadazyma* [45,46]. The lactic acid bacteria (LAB) genera and species, including *Lactobacillus* (*L. casei*, *L. acidophilus*, *L. fermentum*, *L. delbrueckii*, *L. helveticus*, *L. paracasei*, *L. pentosus*, *L. plantarum*, *L. salivarius*, *L. rhamnosus GG*, *L. johnsonii*), *Bifidobacterium* (*B. bifidum*, *B. longum*, *B. lactis*, *B. infantis*, *B. breve*, *B. adolescentis*), *Leuconostoc* spp. (*Ln. lactis*, *Ln. mesenteroides*subsp. *Cremoris*, *Ln. mesenteroid*es subsp. dextranicum), and *Streptococcus* spp. (*S. salivarius* subsp. thermophilus) are highly diversified and studied probiotic bacteria implicated in the inhibition and apoptosis of various human cancer cells [32,47,48,49]. Other reported bacterial probionts include *Bacillus* (*B. fermenticus*, *B. subtilis*), *Clostridium butyricum*, *Enterococcus faecium*, *Pediococcus pentosaceus*, *Lactococcus lactis*, *Propionibacterium*, and *Streptococcus thermophilus* [49]. *Actinomycetes* such as *Streptomyces* and *Micromonosporaceae* are also promising candidates. Trioxacarcins A–C, anthraquinone, Macrodiolide tartrolon D, and Streptokordin obtained from *Streptomyces* spp. exhibited significant antitumor/cytotoxic activities against various cancer cell lines [50,51,52].

**Figure 1:**
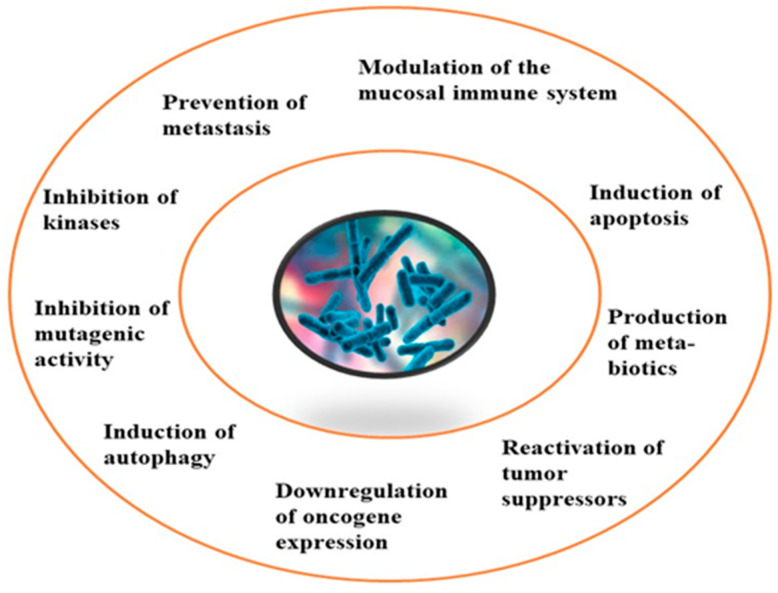
General mechanism of action of cancer prevention and management.

Although limited information is available on cancer management among marine probiotics, few probiotics are known to possess anticancer properties through several mechanisms (Figure 1), primarily due to meta-biotics, which consist of structural components, metabolites, and signaling molecules with specific chemical structures as shown in *Enterococcus lactis* IW5 [53,54,55]. These components optimize various physiological functions of the host, including regulatory, metabolic, and behavioral reactions. Marine probiotics, such as *Lactobacilli* and *Bifidobacteria*, modify the mucosa by increasing the production of chemokines and host defense peptides, inducing dendritic cell maturation, and increasing cell proliferation and apoptosis [51]. Marine probiotics can modulate cancer by inducing apoptosis, inhibiting mutagenic activity, downregulating oncogene expression, inducing autophagy, inhibiting kinases, reactivating tumor suppressors, preventing metastasis, and producing meta-biotics, as already shown in *B. animalis*, *B. infantis*, *B. bifidum*, *L. paracasei*, *L. acidophilus*, and *L. plantarum* I-UL4 against MFC7 cancer cells [53,54]. Although probiotics alone may not suffice in treating cancer, they can mitigate colorectal cancer (CRC) by enhancing the efficacy of treatments and acting on the immune system [56]. Studies have shown that probiotic strains, specifically lactic acid bacteria mixtures, can differentially induce and modulate macrophage pro- and anti-inflammatory cytokines and phagocytosis [53]. It also can mitigate the effects of DMH-induced colon shortening and positively affect leukocyte count and colon tumor growth [56]. The combinations also induce the excretion of proinflammatory IL-18 by tumor cells and are crucial in mitigating CRC [56]. Probiotics generally exhibit antitumor activities by enhancing the intestinal microbiota, degrading possible carcinogens, and modulating gut-associated and systemic immune responses [57]. Worthy of mention is that several anticancer drugs of marine origin are in clinical use with sufficient approvals, including cytarabine, vidarabine, nelarabine, fludarabine phosphate, trabectedin, eribulin mesylate, brentuximab vedotin, polatuzumabvedotin, enfortumabvedotin, belantamabmafodotin, plitidepsin, lurbinectedin, bryostatins, discodermolide, eleutherobin, and sarcodictyin [41,58].

With the increasing popularity of antibiotic stewardship, the misuse and abuse of antimicrobials in developed and developing countries have remained high, with an attendant increase in the development of bacterial resistance. Especially with its forecasted implications [59], possible avenues for developing newer antimicrobials must be exploited. Marine probiotics are a potential source of antimicrobial substances. Pereira et al. [59] showed that marine-isolated *L. lactis* and *E. faecium* produce effective bacteriocin antibiotics [59]. The bacteriocin-producing potential of *Lactococcus* spp. also agrees with Sarika et al.’s study [60]. Other marine probiotics that can produce antibacterial substances have continued to emerge. *Pseudoalteromonas* spp. and *Vibrio* spp. produced antibacterial substances with activity comparable to established antibiotics [61]. Marine lactic acid bacteria of the genera *Lactococcus* spp., *Enterococcus* spp., *Lactobacillus* spp., and *Leuconostoc* spp. have been reported to produce antimicrobial substances against *Vibrio* spp. and *Photobacterium* spp. [62]. Kaktchan et al. [63] revealed that *Lactococcus* spp., cultured in an earthen pond, could produce a bacteriocin-like substance that inhibits the growth of *Vibrio* spp. and *Pseudomonas aeruginosa*.

Immune system arsenals generally recognize viral antigens, preventing their multiplication within the host [64]. However, research should remain proactive, as infective agents constantly change and could sprout newer pathogenic strains. Although the exact mechanisms of action are still unclear, some probiotics have shown a solid ability to prevent viral multiplication in fish and can be used as antiviral agents. *Lactobacillus* spp. and *B. subtilis* boosted viral resistance by preventing viral infections in *Paralychthus olivaceus* and grouper fish, respectively [65,66]. The production of antiviral compounds by *Pseudoalteromonas* spp. has protected Prawns and Sea breams against viral pathogens [67]. Other studies have also shown the possibility and potential of deploying probiotics in the marine environment as agents to prevent viral infections [68,69].

A robust immune system correlates well with defense against infections and diseases. Studies on the immune-enhancing potential of marine probiotics have shown promising results [33,37]. Wasana et al. [47] reported the expression of the immunomodulatory genes in zebrafish larvae exposed to a novel *Pseudoalteromonas xiamenensis*, a marine probiotic that induced the down-regulation of proinflammatory cytokine genes. In another study, a significant reduction in the levels of proinflammatory cytokines was observed in CRC patients who received six viable probiotics of *Lactobacillus* and *Bifidobacterium* strains [70].

The propensity of reactive oxygen and reactive nitrogen species as free radicals to alter the body’s proteins, lipids, and DNA is a significant cause of some human diseases [71]. Due to synthetic antioxidants’ toxic effects [72], marine probiotics have been studied as a natural source of antioxidants. In a study, Alsharmmari et al. [73] espoused the novel marine probiotic, *Enterococcus durans*, which possesses an efficient antioxidant potential. Other studies that corroborated and supported the possible use of marine probiotics as potential sources of antioxidants are those by Husain et al. [73] and Angulo et al. [74].

**Figure 2:**
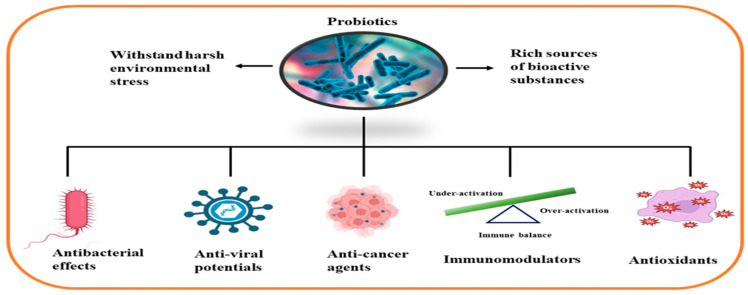
Therapeutic potentials of marine probiotics.

Representative marine probiotic-derived drugs and their microbial sources are presented in Table 2. Therapeutic potentials of marine probiotics are illustrated in Figure 2.

**Table 2:** Representative marine probiotic-derived drugs and their microbial sources.

Bioactivity	Drugs	Microbial Source	Reference
Anticancer	Actinomycin, Salinosporamide A (Marizomib^®^) (NPI-0052), Plinabulin, Enzastaurin, Lestaurtinib, Becatecarin, GSK2857916, Ladiratuzumab vedotin, Tisotumab vedotin, Glembatumumab vedotin, Denintuzumab mafodotin, Midostaurin (Rydapt^®^), Pinatuzumab vedotin, ASG-15ME, Lifastuzumab vedotin, Vandortuzumab vedotin, UCN-01	*Streptomyces* sp., *Salinospora tropica*, *Aspergillus* sp., *Streptomyces staurosporeus*, *Saccharothrix aerocolonigenes*, *Caldora penicillata*	[75,76]
Antimicrobial	Gageotetrins A–C, Gageopeptides A–D, Ieodoglucomide 1, 2, Marinopyrrole A, Merochlorin A, Anthracimycin	*Bacillus subtillis* 109GGC020, *Bacillus licheniformis* 09IDYM23, *Streptomyces* sp.	[77,78]
Immunomodulatory	Brentuximab vedotin, Polatuzumab vedotin (DCDS-4501A), Belantamab madofotin-blmf	*Symploca* sp. VP642, Cyanobacteria	[79]
Antioxidant	Hexaricins F, Asperchalasine I	*Streptosporangium* sp. CGMCC 4.7309, Mycosphaerella sp. SYSU-DZG0	[80]
Antiinflammatory	Cyclic peptide cyclomarin, Violaceomide A, Dehydrocurvularin	*Streptomyces* sp., *Aspergillus terreus* H010, *Penicillium sumatrense*	[78,80]

## 5. *Pseudoalteromonas* spp.; Therapeutic Potentials and Systematic Survey

Of the class *Gammaproteobacteria*; the cold-adapted *Pseudoalteromonas* spp. are widely distributed heterotrophic, flagellated, non-spore-forming, rod-shaped gram-negative marine probiotic bacteria with up to 37 to 48 specified species [70,71,72] proposed and separated from *Alteromonas* as a genus in the last 28 years [81]. The relatively low number of studies on the genus could be attributed to the recency of the genus proposal. In addition, it is difficult to reach and manage these isolates [82]. They are abundant, comprise 20 to 60% of the marine microbial community, and are often associated with particles [83]. They are well known for possessing and releasing a broad range of extracellular bioactive substances, including enzymes, pigments, protease, and polysaccharides, with many biotechnological and pharmaceutical applications [84,85,86]. They are implicated in the cycling of nutrients, including carbon and nitrogen, and in the intestinal microbiota of marine life forms, assisting in maintaining physiological functions [87]. Recent studies have shown the *Pseudoalteromonas* spp. to possess diverse bioactive/therapeutic potentials involving their metabolites, including having anticancer (Table 3) and antibacterial effects (Table 4) via different mechanisms. They are presented here in four categories, including (1) genes and proteins/enzymes, (2) polymers, polysaccharides, and peptides, (3) extracts and organic compounds, and (4) nanoparticles, as studies have been reported in these lines.

**Table 3:** Anticancer activities of *Pseudoalteromonas* spp.

Category	*Pseudoalteromonas* sp.	Study	Method	Active Agent	Structure	Mechanism of Action	References
Proteins/enzymes	*Pseudoalteromonas* sp. Xi13, (genomically associated with *P. carrageenovora* IAM 12662 and *Pseudoalteromonas* sp. An99)	Comparison of immobilized and lyophilized k-Selenocarrageenase produced from *Pseudoalteromonas* sp. Xi13 and The complete genome sequencing of *Pseudoalteromonas* sp. Xi13 and anticancer effects of the selenium oligosaccharides.	Response Surface Methodology, 16S rRNA gene sequencing, and Cell mass detection of cancer cells	k-Selenocarrageenase	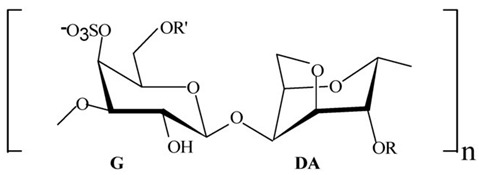	k-Selenocarrageenase acts by degrading k-Selenocarrageenan to selenium oligosaccharides, possibly involving complex glycoside hydrolase	[88,89]
	*P*. *tetradonis*, *P. porphyrae* LL1, *Pseudoalteromonas* sp. ZDY3	Catalytic activity and thermostability enhancement of two similar mutants k-Carrageenases. The expression of the same enzyme from *P. porphyrae* LL1 in *B. choshinensis*	PoPMuSiC algorithm. Cloning, transformation, and enzyme activity assays. Enzyme isolation, purification, LC-HRMS	k-Carrageenases.	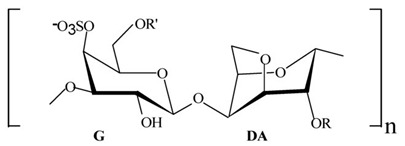	Produces even numbered k-Carrageenan oligosaccharides by catalyzing k-Carrageenans	[90,91,92]
	*P. carrageenovora*	Characterization (pH and temperature) of arylsulfatase from *P. carrageenovora* mutants	Library construction, sequencing, expression, purification, and determination of arylsulfatase activity following pH and temperature variations.	Arylsulfatase	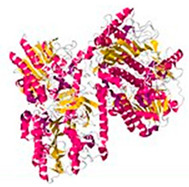	The use in the detection of cancer cells was not stated. However, arylsulfatase enables the arylsulfatase hydrolysis into inorganic sulfate and aryl compounds.	[93]
	*Pseudoalteromonas* sp. strain 1020R	Determined the effects of prodigiosins produced by *Pseudoalteromonas* sp. strain 1020R on the protein kinases and phosphate as targets in the cell death of cancer cells through apoptosis using leukemia cell lines.	Cytotoxicity, protein phosphatase, and kinase assay	Prodigiosins (2-methyl-3-pentylprodiginine, 2-methyl-3-heptylprodiginine, 2-methyl-3-butylprodiginine, and 2-methyl-3-hexylprodiginine)	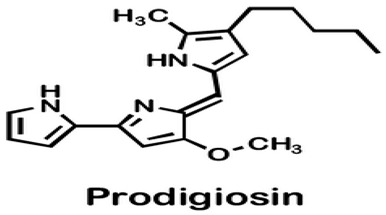	Protein-phosphatase inhibited cytotoxicity of the leukemia cancer cells. Also, that mechanism activation is independent of the p53 protein.	[94]
Polymer, polysaccharides, and peptides	*P. nigrifaciens* Sq02-Rif^T^	Assay of the antitumor effect on colon cancer cells by capsular polysaccharide isolated from *P. nigrifaciens* Sq02-Rif^T^	Chemical and spectroscopic methods, Cell viability assay	Capsular polysaccharide	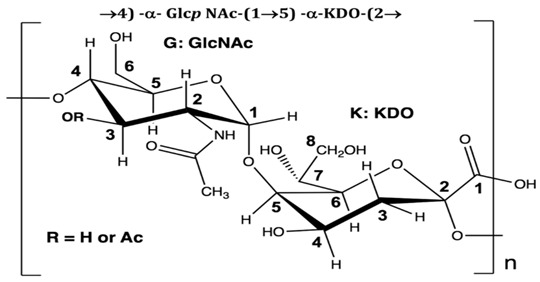	Activate Caspases -3 and -9 on the CaCo-2 and HCT-116 of the cancer cells inducing apoptosis.	[95]
	*P. piscicida* SWA4_PA4.	Iron chelating activity and cytotoxicity effects (against human T lymphocyte cells) of novel Pseudoalteropeptide A (lipopeptide) isolated from *P. piscicida* SWA4_PA4.	Peptide isolation, spectroscopy, and viability assay	Pseudoalteropeptide A	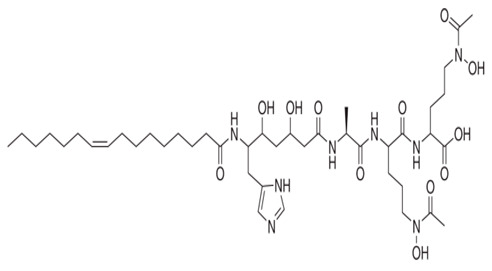	Iron chelating activity and cytotoxicity effects	[96]
Extracts and organic compounds	*P. haloplanktis* TAC125	The antiproliferative effects of ethyl acetate crude extract and compounds of marine *P. haloplanktis* TAC125 against A549 lung epithelial cancer cells	3-(4,5-Dimethylthiazol-2-yl)-2,5-Diphenyltetrazolium Bromide (MTT) viability assay, bioassay-guided purification, and mass spectrometry	4-hydroxybenzoic acid	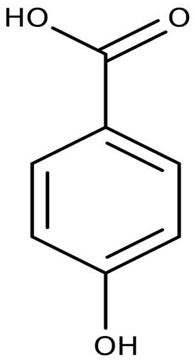	Activates specific well-regulated molecular pathways, Pyroptotic cell death signaling, which triggers the transcription of Caspase -1 and subsequent proinflammatory cytokines	[97]
Nanoparticles	*P. shioyasakiensis*	Reduction and cytotoxicity effects of *P. shioyasakiensis*-based nanoparticles.	Estimation of cell viabilities.	Selenium and tellurium nanoparticles of *P. shioyasakiensis*	-	Different pathways and enzymes, including reductases, siderophores, glutaredoxin, and glutathione, in converting selenium and tellurium to their nanoparticles; however, the specific mechanisms used by the isolates were not covered.	[83]

### 5.1. Marine Probiotic Pseudoalteromonas spp. in the Prevention/Management of Cancer

#### 5.1.1. Anticancer *Pseudoalteromonas* spp. Proteins/Enzymes

Denge et al. [88] compared immobilized and lyophilized k-selenocarrageenase, a potential adjuvant for cancer drugs obtained from *Pseudoalteromonas* sp. Xi13. Although the lyophilized enzyme had a better recovery rate (70%), the immobilized enzyme exhibited better stability after several uses. Mechanistically, k-selenocarrageenase acts by degrading k-selenocarrageenan to selenium oligosaccharides, addressing the issues of water solubility and the high molecular weight of the former [88]. In a study involving the complete genome sequencing of the same isolate, Wang et al. [89] suggested the mechanism of the k-Selenocarrageenase action to involve complex glycoside hydrolase. They also confirmed the anticancer effects of selenium oligosaccharides (specifically, disaccharides and tetrasaccharides) in a concentration-dependent manner in HeLa cervical cancer cells. Hong et al. [90] reported the catalytic activity and thermostability enhancement of two mutant k-carrageenases produced from *P*. *tetradonis*, which produces even numbered k-carrageenan oligosaccharides by catalyzing k-carrageenans via the PoPMuSiC algorithm. Compared to wild-type k-carrageenase, the enzyme mutants displayed better enzyme activity, attributed to better enzyme flexibility and fewer structural deviations. Zhao et al. [91] demonstrated the expression of the same enzyme from *P. porphyrae* LL1 in *Brevibacillus choshinensis*, which also presented considerable stability across a broad pH range. They showed that Mg^2+^ drastically enhanced the enzyme’s activity and that the enzymatic hydrolysates were made of An-G4S-type neocarrabiose units, the end products of which were neo-carratetraose. Also, Zhao et al. [92] isolated stable, high hydrolyzing k-carrageenase from *Pseudoalteromonas* sp. ZDY3 with Km and K_cal_/K_m_ of 3.67 mg/mL and 53 mL/mg/s, respectively. The enzyme end-products were k-neocarratetraose and k-neocarrabiose. Arylsulfatase, which plays a potential role in cancer cell detection, was isolated by Zhu et al. [93] from a library of *P*. *carrageenovora* arylsulfatase mutants, which showed improved thermal and pH stability compared with the wild-type enzyme. Soliev et al. [94] determined the effects of prodigiosin (2-methyl-3-pentylprodiginine, 2-methyl-3-heptylprodiginine, 2-methyl-3-butylprodiginine, and 2-methyl-3-hexylprodiginine) produced by *Pseudoalteromonas* sp. strain 1020R on protein kinases and phosphate as targets of cancer cell death through apoptosis in leukemia cell lines (HL60, K562, and U937). They reported the dose-dependent inhibition of two phosphates, including protein phosphatase 2A and tyrosine phosphatase 1B. However, the prodigiosins were largely inactive against the protein kinases. Thus, they suggested protein–phosphatase inhibition-based cytotoxicity in leukemia cancer cells. Also, the mechanism activation is independent of the p53 protein owing to the low concentration of the prodigiosin in the leukemia cell amidst the apoptosis.

#### 5.1.2. Anticancer *Pseudoalteromonas* spp. Polymer, Polysaccharides, and Peptides

Di Guida et al. [95] reported antitumor effects against colon cancer cells (compared to the untreated cell, reducing 60% of colon cancer cell viabilities at 600 μg/mL after 72 h and with a nontoxic lower cell viability reduction (20%), when compared to the untreated control cells, at 200 μg/mL, after 72 h) by a capsular polysaccharide made up of *N*-acetylated aminosugars, isolated from *P. nigrifaciens* Sq02-Rif^T^ and obtained within a fish gut, which activates Caspases-3 and -9 on the CaCo-2 and HCT-116 of the cancer cells and induces apoptosis. Ueoka et al. [96] showed the iron chelating activity and cytotoxic effects (against human T lymphocyte cells) of a novel Pseudoalteropeptide A (lipopeptide) isolated from *P. piscicida* SWA4_PA4.

#### 5.1.3. Anticancer *Pseudoalteromonas* spp. Extracts and Organic Compounds

The antiproliferative activity of ethyl acetate extract and the bioactive compounds of *P. haloplanktis* TAC125 against A549 lung epithelial cancer cells were investigated by Sannino et al. [97]. 4-hydroxybenzoic acid from the extract was identified as an anticancer cell proliferation (with IC_50_ value ≤ 1 μg/mL) compound in the bacterial extract; it exhibited a specific gene and protein level well-regulated pathway termed pyroptotic cell death signaling, which triggers the transcription of Caspase-1 (an inflammasome) and subsequently the release of IL18β and IL18 encoding genes (proinflammatory cytokines).

**Table 4:** Antibacterial activities of *Pseudoalteromonas* spp.

Category	*Pseudoalteromonas* sp.	Study	Method	Active Agent	Structure	Mechanism of Action	Reference
Genes and proteins/enzymes	*Pseudoalteromonas* sp. 1400	*P. aeruginosa-*implicated biofilm inhibition by alginolytic enzymes produced by thirty-six bacterial isolates, including *Pseudoalteromonas* sp. 1400	Enzyme isolation, activity, antibiofilm assays, immunofluorescent staining, and chromatography	Alginate lyase (AlyP1400)	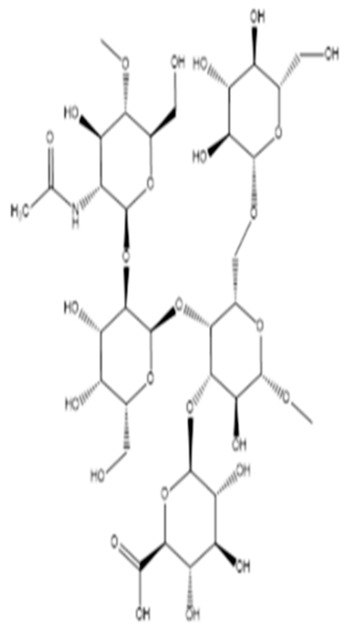	Dual lyase activity of degrading poly-glucuronic and -mannuronic acids. Hydrolytic and disruptive effects of the enzyme against the extracellular alginate produced by *P. aeruginosa* CF27	[98,99]
	*P*. *luteoviolacea* S4054	Assay of the bioactive violacein and indolmycin produced by a mutant *P*. *luteoviolacea* S4054	Antibacterial/diffusion, UHPLC-UV/Vis studies	Violacein, indolmycin	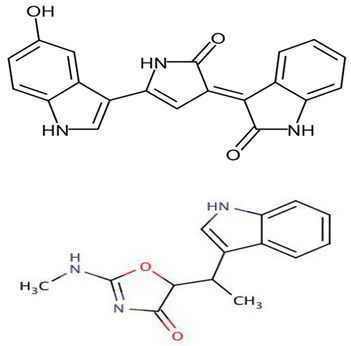	Not available	[100]
	*P. phenolica* KCTC 12086^T^	The complete genome elucidation of *P. phenolica* KCTC 12086^T,^ which could produce antibiotic compounds	Genome sequencing techniques	Polybrominated compounds: polybrominated-diphenyl ethers, -bipyrroles, and -biphenyls	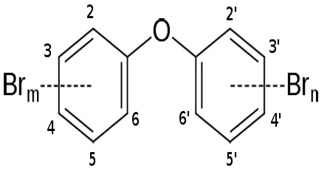	Not available	[101]
	*P. xiamenensis* STKMTI.2	The complete genome elucidation of *P. xiamenensis* STKMTI.2	Genome sequencing techniques	Brominated marine phenols/pyrroles and secondary metabolites (peptides, butyrolactone, prodigiosin, RiPP-like, and Lant I)	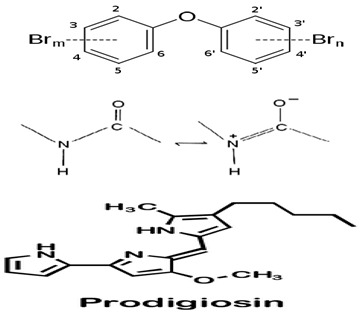	Not available	[102]
	*Pseudoalteromonas* sp. NC201	The complete genome elucidation of *Pseudoalteromonas* sp. NC201	Genome sequencing techniques	Peptides (bacteriocins and gramicidin/tyrocidine)	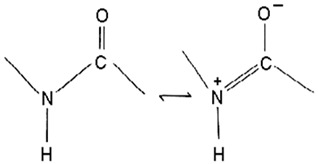	Not available	[103,104]
	*P. tunicate*	Studied and sequenced the tam operon, which initiates the synthesis of *P. tunicate*-produced tambjamine YP1, a natural bipyrrole antibiotic	Genome sequencing techniques, mass spectrometry analysis	Tambjamine YP1	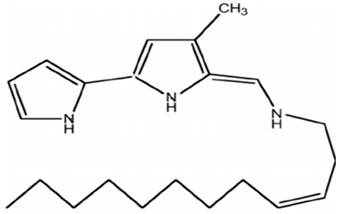	Not available	[105]
	*P. luteoviolacea*	Analyzed different geographic sort isolates of *P. luteoviolacea* for biosynthetic richness and diversity	Metabolomic, Genome sequencing techniques, mass spectrometry analysis	Indolmycin	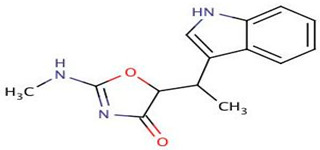	Not available	[106]
	*P. rubra*	Genomic study of *P. rubra* gene responsible for cycloprodiginine production.	Genome sequencing techniques	Cyclo-prodiginine	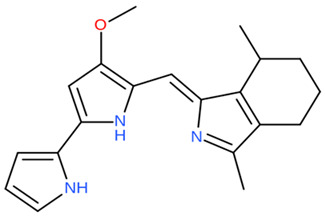	Not available	[107]
	*P. flavipulchra* JG1	Genome sequence of *P. flavipulchra* JG1 responsible for producing *P. flavipulchra* antibacterial Protein (PfaP) for specific genes or clusters responsible for the expression of the antibacterial protein, proven to have inhibitory effects against certain bacteria.	Genome sequencing techniques	*P. flavipulchra* antibacterial Protein (PfaP)	-	*P. flavipulchra* antibacterial Protein (PfaP) oxidatively deaminates certain amino acids to α-keto acids, hydrogen peroxide, and ammonium. Hydrogen peroxide decomposes into other metabolites, which exhibit antimicrobial effects.	[108]
	*P. piscicida*	Genome sequencing of *P. piscicida* 36Y_RITHPW produces bioactive compounds with inhibition effects against multi-resistant *V. parahaemolyticus* implicated in Shrimp hepatopancreatic necrosis disease.	Genome sequencing techniques	Bacteriocins, peptides	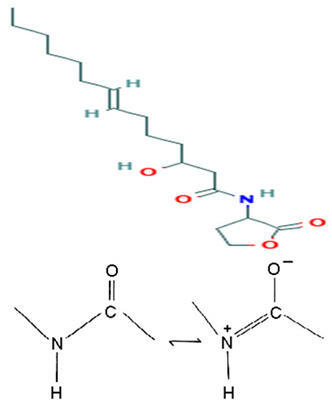	Not available	[109]
	*P. piscicida* strain DE2-B	Genome sequence of *P. piscicida* strain DE2-B	Genome sequencing techniques	Proteolytic enzymes, peptides, polyketides, and alkaloids	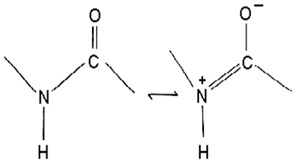	Not availables	[110]
	*Pseudalteromonas* sp. 3J6	Sequence elucidation and antibiofilm effect of *Pseudalteromonas* sp. 3J6	Genome sequencing techniques, Antibiofilm assay	Alterocin	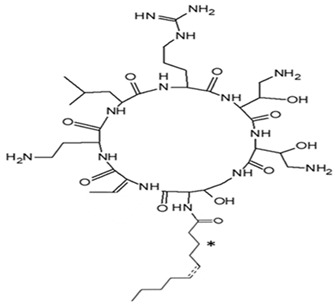	Not available	[111]
Polymer, Polysaccharides, and peptides	*P. prydzensis/*mariniglutinosa, *P. paragorgicola/elyakovii*	Isolation, identification of peptides and bacteria of Oysters hemolymph and their antibacterial effects.	Antimicrobial activities	Peptides	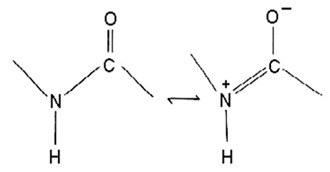	Not available	[112]
	*Pseudoalteromonas* spp (Designgated hCg-6, hCg-42, hOe-66, hOe-124, and hOe-125)	Alterins isolated using the bacterial cell-free culture were subjected to antibacterial assays against some gram-negative bacteria. Mechanism of alterin activity determined via lipopolysaccharide binding assays	Minimal Inhibitory Concentration (MIC), UPLC-HRMS	Alterins	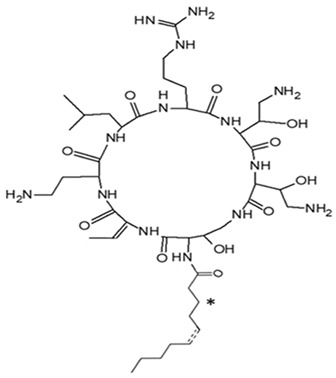	Provide the depolarization of the bacterial membrane and the subsequent cell permeabilization and lysis.	[113,114]
	*P. shioyasakiensis* and *P. mariniglutinosa*.	Poly-hydroxybutyrate-co hydroxy-hexanoate (PHBH) degrading abilities of certain gram-negative bacteria, including *P. shioyasakiensis* and *P. mariniglutinosa*.	Bacterial isolation, degradation, and isolation of monomers, antibacterial assay	3-hydroxy-butyrate, 3-hydroxy-hexanoate, hydroxy-alkanoic acids	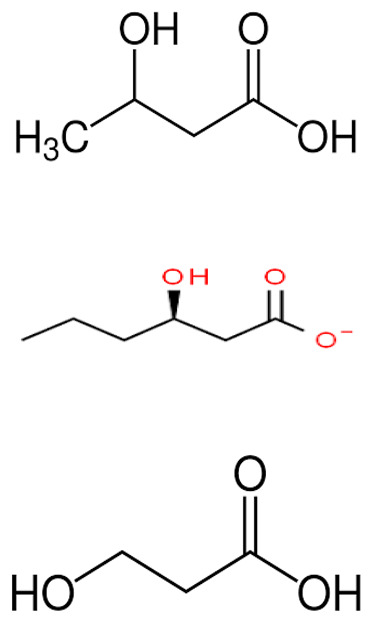	Degradation of Poly-hydroxybutyrate-co hydroxy-hexanoate (PHBH) to antibacterial monomers	[115]
	*Pseudoalteromonas* sp. SANK 71903	Tested an array of secondary metabolites of *Pseudoalteromonas* sp. SANK 71903 for potential inhibition effects against lipopolysaccharide (LPS)	LPS, inhibition assay, MIC	Cyclic Peptides, Ogipeptins (A–D)	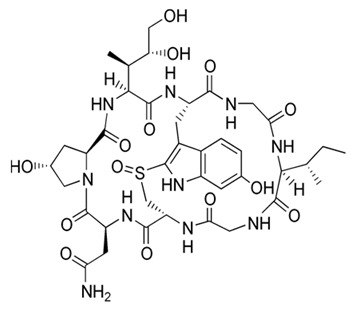	LPS inhibition by CD14 binding and cytokine secretion blockage from LPS-stimulated cells.	[116]
Extracts and organic compounds	*P. ruthenica* S6031.	In-vitro and vivo studies by Wasana et al. [117] demonstrated the probiotic potentials of *P. ruthenica* S6031, especially against *E. piscicida*.	Agar spot, In vivo studies	*P. ruthenica* S6031 supplements	-	Not available	[117]
	*Pseudoalteromonas* sp. J010	Determination of the antibacterial effect of korormicin against *Vibrio* sp.	MIC, sequence data studies	Korormicin	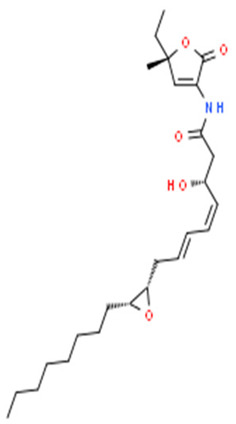	Korormicin releases reactive O_2_ species via the electron transfer initiation in the enzyme, which promotes O_2_ and some redox cofactors reactions.	[118]
	*Pseudoalteromonas* spp. coded CDM8 and CDA22.	In vitro antibacterial activity and in vivo probiotic potential against *V. parahaemolyticus*, and PCR evaluations of two *Pseudoaltermonas* spp.	In vitro, in vivo, PCR evaluations	*Pseudoalteromonas* spp. coded CDM8 and CDA22	-	Reduction of the copy number of *V. parahaemolyticus* toxin production gene, PirA^vp^	[119]
	*P. flavipulchra* CDM8	Described the antibacterial mechanisms of *P. flavipulchra* CDM8 with potent inhibition activities against *Bacillus* spp. and *Vibrio* spp.	Antimicrobial biofilm assays and microscopy	Hydrogen peroxide, PfaP-like antibacterial proteins, and other molecules, surface positioned vesicle/pilus-like structure	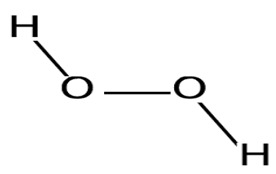	The transferable outer surface positioned vesicle/pilus-like structure likely contributes to the inhibition activities observed and is described as a novel mechanism of antibacterial activity by the isolate.	[120]
	*Pseudoalteromonas* sp. type strain S4498	Identification and determination of the mechanisms of secondary metabolites of *Pseudoalteromonas* sp. type strain S4498	Use of antiSMASH and bioassays	Tetrabromopyrrole	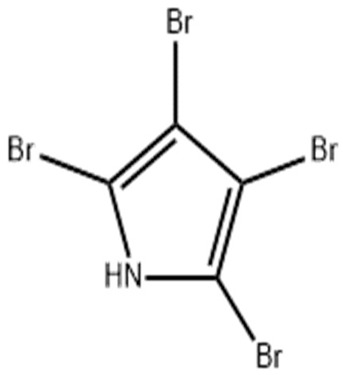	Tetrabromopyrrole induces its antimicrobial effects through its signaling properties, induction of cellular stress, and the activities of dibromo-maleimide, its oxidized by-product.	[121]
	*Pseudoalteromonas* sp. (designated as hCg-51)	Assay of the different cell-free cultures of 843 species/strains obtained within the haemolymphs microbiota of bivalve species from the sea	Well diffusion and hemolymph-associated strain assays	*Pseudoalteromonas* sp. (designated as hCg-51)	-	Not available	[122]
	*Pseudolateromonas* spp., *Pseudolateromonas* sp. (hCg-6)	Assay of a collection of 11 *Pseudolateromonas* spp. obtained from the hemolymph of mussels and oysters for their antibacterial activities against *V. harveyi*.	Well diffusion, PCR, and hemolymph-associated strain assays	*Pseudolateromonas* spp., *Pseudolateromonas* sp. (hCg-6)	-	Not available	[123]
	*P. issachenkonii*, *P. haloplanktis* TAC125	Antibacterial activities against *E. coli*, *S. epidermis*, *and K. rhizophila* by solvents extracts two *Pseudoalteromonas* spp., among other marine bacteria isolated from the surface of various marine macroalgae.	Well diffusion assay and phylogenetics	*P. issachenkonii*, *P. haloplanktis* TAC125	-	Not available	[124]
	*P. haloplanktis* TAC125	Antibiofilm activity of the culture supernatant of *P. haloplanktis* TAC125 against *S. epidermidis*	Antibiofilm assay	*P. haloplanktis* TAC125	-	Disruption of the *S. epidermidis* biofilm multicellular structure.	[125]
	*P. haloplanktis* TAC125	Culture, fermentation, and antibacterial activity of methylamine from *P. haloplanktis* TAC125	Fermentation, minimum volatile inhibitory concentrations	Methylamine	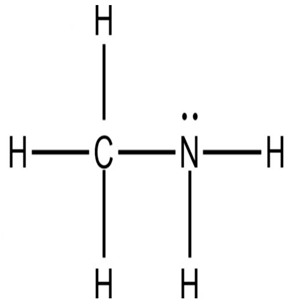	Sannino et al., via a defined culture and fermentation system, produced and accumulated methylamine, a volatile organic compound (VOC), from *P. haloplanktis* TAC125, which by the minimum volatile inhibitory concentrations, presented a dose-dependent inhibition of different strains of *Burkholderia* spp. and *E. coli*.	[126]
	*P*. *haloplanktis*	Assay of the antibacterial properties of pentadecanol, a metabolite of *P*. *haloplanktis*, and the derivatives against *Listeria monocytogenes*	Minimum inhibitory concentrations.	Pentadecanol	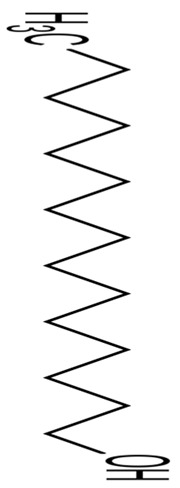	Not available	[127]
	*P*. *haloplanktis* TAC125	Antibiofilm activity against *S. epidermis* O-47, *S. epidermidis* RP62A	Antibiofilm assay	Pentadecanol	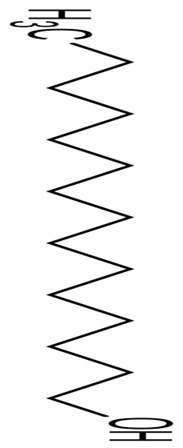	The interference of the quorum sensing system of the *S. epidermis* by the AI-2 signaling process.	[128]
	*P. rubra* TKJD 22	Isolation of organic compounds from Tunicate-associated bacteria and antibacterial activities of compounds.	Sequencing, NMR, Minimum inhibitory concentration	Isatin	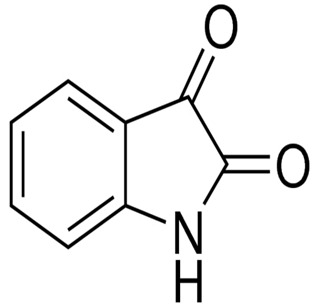	Not available	[129]
	*Pseudoalteromonas* strain J010	Isolation of bioactive compounds from the ethanol extract of *Pseudoalteromonas* strain J010 and determination of their antimicrobial activities.	Disc-diffusion assay, MS, and NMR techniques.	4′-(3,4,5-tribromo-1 *H*-pyrrol-2-yl) methyl) phenol (a bromopyrrole), 5 koromicins, and tetrabromopyrrole	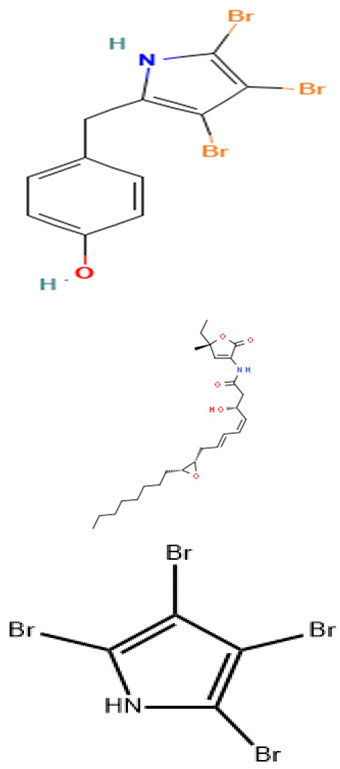	Not available	[130]
	*P. piscicida* S2040	Isolation of bioactive compounds from the extracts of *P. piscicida* S2040 and determination of their antimicrobial activities.	Disc-diffusion assay, MS, and NMR techniques.	Bromo- and dibromoalterochromides, myxochelins A and B, and alteramide A	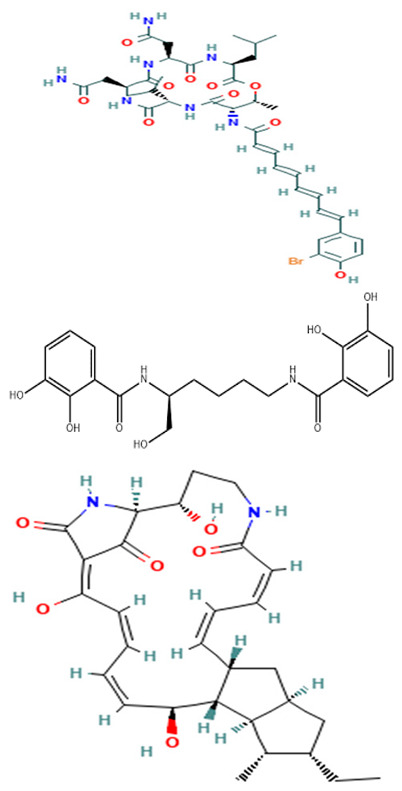	Not available	[131]
	*P. piscicida* 2202	Antibacterial activity of *P. piscicida* 2202 isolated from *Modiolus kurillenis*	Antimicrobial activity	*P. piscicida* 2202	-	Not available	[132]
	*Pseudoalteromonas* sp. JS19 MT102924.1	Extraction, phytochemical assay, and antibacterial effect of *Pseudoalteromonas* sp. JS19 MT102924.1	Antimicrobial activity, FTIR	*Pseudoalteromonas* sp. JS19 MT102924.1	-	Not available	[133]
Nanoparticles	*P. shioyasakiensis*	Antimicrobial effects of *P. shioyasakiensis*-based nanoparticles	Inhibitory zones and minimum inhibitory concentrations	Selenium and tellurium nanoparticles of *P. shioyasakiensis*	-	The study suggests the contribution of different pathways and different enzymes, including reductases, siderophores, glutaredoxin, and glutathione, in the conversion of selenium and tellurium to their nanoparticles; however, the particular mechanisms used by the isolates were not covered.	[83]

NB: ‘*’ in the Alterocin/Alterin structure is hydroxylation at C_3_ position.

#### 5.1.4. Anticancer *Pseudoalteromonas* Nanoparticles

Beleneva et al. [83] used a green technology to synthesize the nanoparticles of selenium and tellurium using three different isolates of *P. shioyasakiensis* from a marine test facility in Vietnam, which presented high reduction process effects and human breast cancer and dermal fibroblasts cytotoxicity effects, with the selenium-based nanoparticles of the isolates outperforming the tellurium-based nanoparticles.

### 5.2. Antibacterial Activities of Pseudoalteromonas *spp.*

#### 5.2.1. Antibacterial *Pseudoalteromonas* spp. Genes and Proteins/Enzymes

Daboor et al. [98] demonstrated the *P. aeruginosa*-implicated biofilm inhibition by alginolytic enzymes produced by thirty-six bacterial isolates, including *Pseudoalteromonas* sp. 1400, which yielded alginate lyase and had the highest alginolytic activity. The purified enzyme had dual lyase activity of degrading poly-glucuronic and -mannuronic acids and had significant combined antibiofilm activities with carbenicillin and ciprofloxacin, reducing the *P. aeruginosa* biofilms’ surface area, biovolume, and thickness. In a follow-up investigation, using immunofluorescent staining and chromatography, Daboor et al. [99] confirmed the antibiofilm-enhancing effects of the alginate lyase enzyme, AlyP1400, against *P. aeruginosa* CF2F-implicated biofilm. Also, the hydrolytic and disruptive impact of the enzyme against the extracellular alginate produced by *P. aeruginosa* CF27 is reported to be instrumental to the anti-biofilm’s activities. Thogersen et al. [100] assayed the bioactive violacein and indolmycin produced by a mutant *P*. *luteoviolacea* S4054, which had inhibition activities against *Staphylococcus aureus* and *Vibrio anguillarum*. The UHPLC-UV/Vis studies utilized in comparing the expression of the bioactive compounds with those produced by a wild strain revealed a lower and higher production of violacein and indolmycin, respectively, compared to the wild strain. The study concluded that although the significant antibacterial compounds of *Pseudoalteromonas* sp. are yet to be identified, the two bioactive compounds also contribute to the observed antibacterial properties.

The complete genome elucidation of *P. phenolica* KCTC 12086^T^, which could produce antibiotic compounds including polybrominated-diphenyl ethers, -bipyrroles, and -biphenyls, with proven antibacterial effects against certain bacteria, including methicillin-resistant *S. aureus*, *E. faecium*, *Enterococcus seriolicida*, and *Enterococcus faecalis* has been reported by Choe et al. [101]. The result showed the presence of two 4,868,993 bp chromosomes with 4,264,659 bp coding regions, which encode a total of 4168 proteins. Also, 28 rRNA (9 operons), six ncRNAs, 113 tRNAs, eight pseudo-genes, and one tmRNA were detected. Furthermore, the bmp cluster (1–10), responsible for producing polybrominated compounds, was observed at different nucleotide positions on the chromosome. Other hydrolysis enzyme coding regions reported, including collagenases, phytase, chitinases, and proteases detected, further support the potential applicability of the isolate. Similarly, the genome of *P. xiamenensis* STKMTI.2 isolated from a mangrove soil sediment in Indonesia was elucidated by Handayani et al. [102], which consisted of 4,563,326 bp with a GC content of 43.2%, two circular and linear plasmids, one chromosome, 25 rRNAs, four ncRNAs, 4824 coding sequences, and a CRISPR. The CRISPR gene detected was attributed to the production of brominated marine phenols/pyrroles and secondary metabolites, including bmp 8 and 9 and peptides, butyrolactone, prodigiosin, RiPP-like, and Lant I, and justified the suspicion of *P. xiamenensis* STKMTI.2 for generating broad-spectrum antimicrobial compounds. The genome of *Pseudoalteromonas* sp. NC201 (from the coastal area of New Caledonia) with antibacterial potentials and has already been assessed for its probiotic effects through enhancement of survival rates in *Litopenaeus stylirostris* infected with *Vibrio nigripulchritudo* [103] was also sequenced by Sorieul et al. [104]. The analysis revealed 115 contigs (>100 bp). Of the contigs, 65 presented six scaffolds with an approximate GC content of 43.25%; two were the largest and represented 4.13 Mbp chromosome and 1.24 Mbp chromid, respectively. The remaining sequences comprised insertion sequences and ribosomal RNA operons in repetitive regions and clusters. These results indicated the ability of the isolate to synthesize antibacterial peptides, including bacteriocins and gramicidin/tyrocidine. In addition, the *lodA* and *lodB* genes, which are related to amino acid oxidases, suggested the production of oxygen peroxide, which can have bacterial inhibitory effects. Marchetti et al. [105] studied and sequenced the *tam* operon, which initiates the synthesis of *P. tunicate-*produced tambjamine YP1, a natural bipyrrole antibiotic, and reported to possess other bioactive potentials. They described the *tam* operon as possessing 19 genes, comprising a fused C-terminal acyl carrier protein and N-terminal adenylation domain, bound C 11 and 12 acyl-adenylate intermediates, transfers chain length of fatty acids from C6–C13 to an isolated acyl carrier protein domain, and thus shows the initiations of the production of tambjamine YP1 via the linkage of the pyrrole and fatty acid pathways. Maansson et al. [106] analyzed different geographic sort isolates of *P. luteoviolacea* for biosynthetic richness and diversity using metabolomic and sequencing techniques. The study reported enormous diversity in the 13 analyzed isolates, with only 2 and 7% of the chemical features and biosynthetic genes, respectively, common in the isolates. Genome sequencing of the isolates revealed the presence of biosynthetic clusters that were attributed to the generation of indolmycin, an antibacterial compound. Rond et al. [107] studied *P. rubra*, a cyclized prodiginine-producing bacterium. The genome sequence revealed an unclustered gene responsible for the enzyme that catalyzes regiospecific C-H and prodigiosin cyclization to cyclo-prodigiosin. Prodiginines are natural products with potent bioactive properties, including antibacterial and anticancer effects. Yu et al. [108] sequenced *P. flavipulchra* JG1, responsible for producing *Pseudoalteromonas flavipulchra* antibacterial protein (PfaP) for specific genes or clusters responsible for the expression of the antibacterial protein, which has been proven to have inhibitory effects against certain bacteria, including *Bacillus* spp., *Aeromonas* spp., and *Vibrio* spp. The isolate also produced p-hydroxybenzoic acid, trans-cinnamic acid, N-hydroxybenzoisoxazolone, 6-bromoindolyl-3-acetic acid, and 2′-deoxyadenosine, with inhibitory effects against some microorganisms, including the previously mentioned bacterial species. The results showed that *P. flavipulchra* JG1 had a total of 5,565,361 bp with a GC content and open reading frames of 43.23% and 4913. The tandem repeats, transposons, and insert sequences were 180, 143, and 5, respectively. It possesses a complex system of genes belonging to ABC-type antimicrobial peptide transport and siderophore export systems, penicillin-binding and beta-lactamase class C proteins, and efflux pumps, all contributing to the generation of bioactive metabolites. Specifically, PfaP, the reported most potent bioactive metabolite of the isolate, oxidizes certain amino acids to α-keto acids, hydrogen peroxide, and ammonium. Hydrogen peroxide decomposes into other metabolites, which exhibit antimicrobial effects. Diaz et al. [109] also sequenced *P. piscicida* 36Y_RITHPW, which produces bioactive compounds with inhibition effects against multi-resistant *Vibrio parahaemolyticus* implicated in shrimp hepatopancreatic necrosis disease. Summarily, 4548, 4217, and 71 genes, protein-coding sequences, and RNA sequences were reported. In addition to other findings, the authors elucidated the expression of bacteriocins and peptides (ribosomally produced and responsible for antibacterial properties) by 12 genes in a cluster, including polyketide synthase/non-ribosomal peptide synthase (PKS/NRPS), lantipeptide gene, type 1 PKS, 7 NRPS, and aryl-polyene/NRPS hybrid clusters. In agreement, the genome sequence of *P. piscicida* strain DE2-B by Richard et al. [110] revealed relatively the same genes responsible for the production of antimicrobial proteolytic enzymes and compounding, including peptides, polyketides, and alkaloids; thus, the *P. piscicida* strain DE2-B’s ability to inhibit some bacteria, including, *V. parahaemolyticus*. Jouault et al. [111] sequenced *Pseudalteromonas* sp. 3J6 and identified gene sequences, including the *alt* gene, which encodes 139 residue proteins and is responsible for the expression of alterocin, a protein implicated in inhibiting *P. aeruginosa* biofilms. Generally, the genome, GC content, and coding region of the *Pseudalteromonas* sp. 3J6 were approximately 4.6 Mb, 39.93%, and 3789, respectively.

#### 5.2.2. Antibacterial *Pseudoalteromonas* spp. Polymer, Polysaccharides, and Peptides

On the isolation, identification, and bacterial origin suspicion of low molecular weight antimicrobial peptides of oyster hemolymph, Defer et al. [112] assayed the culture supernatant of different bacteria resident in the hemolymph for their antibacterial effects. Three strains of *Pseudoalteromonas* spp., designated hCg-6, hCg-10, and hCg-42, with sequence results suspecting the first two to be *P. prydzensis/mariniglutinosa* and the last to be *P. paragorgicola/elyakovii*, displayed ranging antibacterial activities against *Aeromonas hydrophila* CIP 7614, *Listonella anguillarum* NCBIM 829, *Yersinia ruckeri* ATCC 29473, *Bacillus megaterium* ATCC 10778, *Lactococcus garviae* ATCC 43921, and *Salmonella enterica* CIP 829. Two of the strains (hCg-6 and hCg-42) also showed BLIS-production abilities. Desriac et al. [113] showed the antibacterial activity of seven alterins with ranging molecular masses (924–982 Da) extracted from two *Pseudoalteromonas* spp. (hCg-6 and hCg-42) of healthy hemolymph microbiota of *Crassostrea gigas* (oyster) against several gram-negative bacteria, including *Vibrio harveyi* ORM4 and *V. parahaemolyticus* 13-028A/3. The produced alterins belong to the family of cationic cyclo-lipopeptides, which bind to bacterial lipopolysaccharides, provoking membrane depolarization and subsequent cell permeability and lysis [113]. Similarly, Offret et al. [114] obtained alterins from a group of 5 *Pseudoalteromonas* spp. isolated from the oyster hemolymph and also demonstrated ranging antibacterial activities against *E. coli* ATCC 25922, *V. harveyi* OMM4, *V. pectenicida* CIP 105190, *V. tasmaniensis* LGP32, and *V. tapetis* CECT 4600. These studies suggest that alterins can be potent lipopolysaccharide-neutralizing and synergistic antibiotic peptides. The polymer poly-hydroxybutyrate-co-hydroxy-hexanoate (PHBH), through its degradation products, including 3-hydroxy-butyrate and -hexanoate and hydroxy-alkanoic acids, is reported to have antibacterial activities. A review of a specific study found that it inhibits *Vibrio penaeicida* and increases the survival rate of shrimp exposed to *V. penaeicida* following consumption of PHBH-supplemented feed [115]. However, the study [108] reported the high PHBH degrading abilities of certain gram-negative bacteria, including *P. shioyasakiensis* and *P. mariniglutinosa*. This degradation action showed inhibitory/suppression effects against *V. penaeicida* through the generation of antibacterial monomer products, thus suggesting the potential application in aquaculture for the protection of shrimps against infection by *V. penaeicida* through diet supplementation with PHBH and the corresponding implicated PHBH-degrading *P. shioyasakiensis* and *P. mariniglutinosa*. No inhibitory effects were observed when *V. penaeicida* was challenged with *Pseudoalteromonas* spp., without PHBH. Kozuma et al. [116] tested an array of secondary metabolites of *Pseudoalteromonas* sp. SANK 71903 has potential inhibitory effects against lipopolysaccharides (LPS) and typical LPS-bearing bacteria. Cyclic Peptides, Ogipeptins (A–D), closely related to Polymyxin B, were identified and showed the ability to inhibit LPS through CD14 binding with 1C_50_ values of Ogipeptin-A = 4.8 nm, -B = 6.0 nm, -C = 4.1 nm, and -D = 5.6 nm and blockage of cytokine secretion from LPS-stimulated cells. They showed antibacterial effects against E. *coli*, with minimum inhibitory concentrations ranging from 0.25–1.0 μg/mL.

#### 5.2.3. Antibacterial *Pseudoalteromonas* spp. Extracts and Organic Compounds

In vitro and in vivo studies by Wasana et al. [117] demonstrated the probiotic potential of *P. ruthenica* S6031. Broth culture spots of the isolate on microbial lawns of *P. aeruginosa*, *Edwardsiella piscicida*, *A. hydrophila*, and *Candida albicans* revealed significant positive inhibition against *E. piscicida* and *A. hydrophila.* In *E. piscicida-*challenged Zebra fish, there was higher cumulative per cent survival of the animals exposed to *P. ruthenica*-supplemented feed, outperforming the control group without supplements. It also revealed the induction of several immune-stress response gene transcripts and the downregulation of the proinflammatory genes. Thus, the study showed that the treatment group animals had better disease tolerance than the control group, making *P. ruthenica* S6031 a good potential probiotic isolate. The organic compound, korormicin, produced by many *Pseudoalteromonas* spp., including *Pseudoalteromonas* strain J010, is acknowledged by Maynard et al. [118] to have antibacterial activities, including against *Vibrio* spp., *Aliivibrio* sp, and *Pseudomonas* sp. They, however, through MICs and DNA sequence data, disclosed that, though korormicin is a potent inhibitor of NA^+^-pumping NADH (quinone oxidoreductase), the antibacterial effects are not due to the inhibition of the stated enzyme, but to the release of reactive O_2_ species via the electron transfer initiation in the enzyme, which promotes O_2_ and some redox cofactor’s reaction. In vitro, in vivo, and PCR evaluations by Wang et al. [119] showed antibacterial activities against *V. parahaemolyticus*, respective enhancement and decrease of the survival rates of *V. parahaemolyticus*-infected shrimps following exposure to *Pseudoaltermona*s spp-supplemented feed and shrimp hindgut presumptive *Vibrio* sp. counts, and reduced the copy number of *V. parahaemolyticus* toxin production gene, PirA^vp^, respectively, by two *Pseudoalteromonas* spp. coded CDM8 and CDA22. Following proper identification, Wang et al. [120] described the antibacterial mechanisms of one of the isolates, *P. flavipulchra* CDM8, which also showed potent inhibition activities against *Bacillus* spp. and *Vibrio* spp. Using antimicrobial biofilm assays and microscopy, they showed its broad-spectrum antimicrobial activity against both gram-positive and -negative bacteria using filter-impregnated culture from the isolate. They described the mechanism to involve *P. flavipulchra* CDM8 metabolites, including hydrogen peroxide, PfaP-like antibacterial proteins, and other molecules. They also identified a transferable outer surface-positioned vesicle/pilus-like structure, which likely contributes to the observed inhibition activities and is described as a novel mechanism of antibacterial activity by the isolate. Using several genome analysis tools, including the antiSMASH (a secondary metabolite prediction tool) and bioassays, Paulsen et al. [121] identified, among others, a highly halogenated tetrabromopyrrole as the main antibacterial metabolite of *Pseudoalteromonas* sp. type strain S4498. Genome studies revealed that the strain had a genome size of 5.4 Mb and a GC content of 43%. However, they suggested that tetrabromopyrrole induced its antimicrobial effects through its signaling properties, cellular stress induction, and dibromo maleimide activity, the oxidized by-product. Desriac et al. [122], in an assay of the different cell-free cultures of 843 species/strains obtained within the haemolymphs microbiota of bivalve species from the sea, showed the *Pseudoalteromonas* strains to possess the most potent antimicrobial abilities (against 12 marine gram-positive and -negative pathogens), with one *Pseudoalteromonas* sp. (designated as hCg-51) beating the other isolates with inhibition at an extreme dilution of 1:1024. They also presented a dose-dependent beneficial effect of the strain on the hemocyte survival rates. Thus, suggesting their strong probiotic potential. Similarly, Offret et al. [123] assayed a collection of 11 *Pseudolateromonas* spp. obtained from the hemolymph of mussels and oysters for their antibacterial activities against *V. harveyi*. The results showed that more than half of the isolates (54%) had varying inhibition activities, with one coded hCg-6 outperforming the others in the collection, with an impressive inhibition zone of 19 mm. The *Pseudoalteromons* sp. (hCg-6) also revealed significant probiotic activities following the increment in the survival rate of Abalone exposed to hCg-6 and subsequently infected with *V. harveyi*. Also, Tangestani et al. [124] reported antibacterial activities against *E. coli*, *Staphylococcus epidermis*, *and Kocuria rhizophila* by solvent extracts of two *Pseudoalteromonas* spp., among other marine bacteria isolated from the surface of various marine macroalgae. The two relevant isolates were phylogenetically confirmed to be *P. issachenkonii* and *P. haloplanktis* TAC125, associated with the seaweeds *Splachnidium rugosum*, and *Carpophyllum maschalocarpum*, respectively. Papa et al. [125] reported the antibiofilm activity of the culture supernatant of *P. haloplanktis* TAC125 against *S. epidermidis* by disrupting the *S. epidermidis* biofilm multicellular structure. Sannino et al. [126], via a defined culture and fermentation system, produced and accumulated methylamine, a volatile organic compound (VOC), from *P. haloplanktis* TAC125, which by the minimum volatile inhibitory concentrations, presented a dose-dependent inhibition of different strains of *Burkholderia* spp. and *E. coli*. Venuti et al. [127] assayed the antibacterial properties of pentadecanol, a metabolite of *P*. *haloplanktis*, and the derivatives against *Listeria monocytogenes*, using the minimum inhibitory concentrations. The results showed that the derivatives, including the ester, acetal, and carboxylic acid, had no inhibitory effects against the test isolates; however, pentadecanol showed potent antibacterial properties and exhibited a MIC of 0.6 mg/mL. Similarly, Casillo et al. [128] isolated pentadacanol from the same *P*. *haloplanktis*, and they demonstrated its antibiofilm activity against *S. epidermis*. They suggested the mechanism of action of inhibiting the biofilm from involving the interference of the quorum sensing system of the *S. epidermis* through the AI-2 signaling process. Using 16S RNA sequencing and NMR elucidation, an organic compound was obtained and determined to be isatin, respectively, from *P. rubra* TKJD 22 associated with marine tunicates by Ayuningrum et al. [129]. The isatin demonstrated inhibition effects against both the laboratory-isolated and MDR-ESBL *E. coli.* Tebben et al. [130] isolated bioactive compounds from the ethanol extract of *Pseudoalteromonas* strain J010, which were elucidated using MS and NMR techniques. Among others, novel 4′-(3,4,5-tribromo-1*H*-pyrrol-2-yl) methyl)phenol (a bromopyrrole), 5 koromicins, and tetrabromopyrrole were identified and presented ranging antibacterial activities against *S. aureus* and other gram-negative bacteria, including *Vibrio* spp., *P. aeruginosa*, *Pseudoalteromonas* spp., and *Shewanella aquimarina*. In another study, following the inhibition activities of the crude extract fractions of *Pseudoalteromonas piscicida* S2040 against *P. aeruginosa*, Sonnenschein et al. [131] isolated bromo- and dibromoalterochromides, myxochelins A and B, and alteramide A, chosen for their antibacterial effects. Eliseikina et al. [132] isolated *Pseudoalteromonas piscicida* 2202, a natural flora of *Modiolus kurillenis*, which had selective antimicrobial activity against *S. aureus*, *C. albicans*, *and B. subtilis* and no significant activity against *E. coli* and *P. aeruginosa*. They, however, suggested caution in the application as potent probiotic agent. Among other heterotrophic bacteria, Setiaji et al. [133] employed *Pseudoalteromonas* sp. JS19 MT102924.1 in an ethyl acetate extraction system to yield secondary metabolites, through which the phytochemical analysis showed the presence of alkanes, carbonyls, alcohols, and alkenes. The extract had antibacterial activities with inhibition zones of 9.8, 10.8, and 9.8 mm against *A. hydrophilia*, *Vibrio alginolyticus*, and *P. aeruginosa*, respectively.

#### 5.2.4. Antibacterial *Pseudoalteromonas* spp. Nanoparticles

Beleneva et al. [83] reported high concentration (500 and 1000 μg/mL) antimicrobial effects of the selenium and tellurium-based nanoparticles of the isolates of *P. shioyasakiensis* against typed cultures of *E. coli*, *P. aeruginosa*, *S. aureus*, *B. subtilis*, and additionally a fungus (*C. albicans*), with the tellurium-based nanoparticles having overall more significant effects.

## 6. Discussion

**Figure 3:**
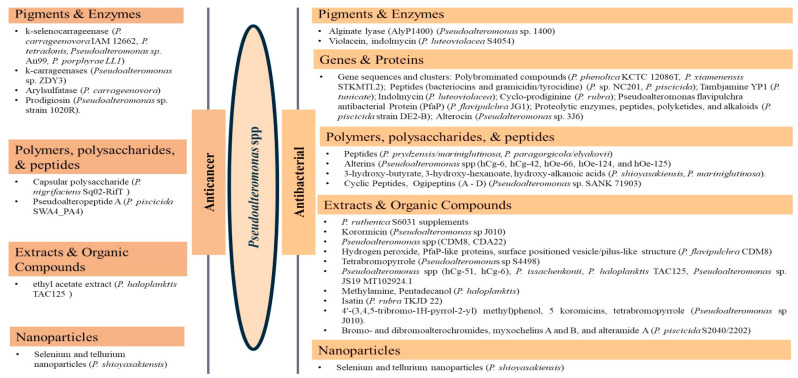
A pictorial presentation of the anticancer and antibacterial constituents of *Pseudoalteromonas* spp.

The review revealed that none of the literature evaluated the genetic basis for the anticancer properties of *Pseudoalteromonas* spp. However, involved k-selenocarrageenases/k-carrageenans, arylsulfatase, and prodigiosin as the pigments and enzymes, capsular polysaccharides, and polysaccharides and Pseudoalteropeptide A in the polymer, polysaccharide, and peptide category, a crude extract of *P. haloplanktis*, selenium, and tellurium nanoparticles in the reported anticancer activities. The reported antibacterial properties involved the production of bioactive pigments and enzymes, including alginate lyase (AlyP1400), violacein, and indolmycin, specific gene sequences and clusters responsible for the expression and production of polybrominated compounds, peptides, polyketides, alkaloids, tambjamine YP1, cylco-prodigine, PFaP, alterocins/alterins, and other compounds including 3-hydroxy-butyrate, 3-hydroxy-hexanoate, hydroxy-alkanoic acids, korormicin, hydrogen peroxide, tetrabromopyrrole, methylamine, isatin, brominated compounds, and nanoparticles by representative tests *Psesudoaltromonas* spp. Much of the literature summarized in Figure 3, however, is currently in the preliminary stages; without an in-depth analysis of the mechanistic and physiological basis of the observed bioactive properties as such, the current review provided the basis and a standpoint for the advancement of the studies including the isolation and extensive characterization of the bioactive compounds with a view to possible clinical application and commercialization.

## 7. Production and Development of Marine Probiotics

Marine probiotics have proven therapeutic applications; however, adequate exploitation and utilization of these properties hinge on optimal industrial-scale production and development. These involve several steps, including isolation, identification, and characterization of potential probiotic strains and testing their efficacy and safety in various applications [134,135]. The first step in marine probiotics production is isolating and identifying likely probiotic strains from marine environments and involves collecting samples from marine ecosystems, such as seawater, sediments, and aquatic organisms and isolating potential probiotic strains using standard microbiological and molecular techniques. The identified strains are then characterized for their morphology, physiology, and biochemistry to profile their potential as probiotics [135]. They are screened and selected for their probiotic properties, including their ability to survive in the host gut system, produce anticancer and antimicrobial compounds, or stimulate the host’s immune system [136]. The selected strains are tested for their safety and efficacy in various applications [137], after which they are mass-produced. Marine probiotics can be produced using different techniques such as batch, fed-batch, and continuous cultures [138,139]. The production process involves growing the selected strains in a suitable growth medium and harvesting and processing the cells to obtain the final product [137,140]. The produced marine probiotics are formulated into an appropriate delivery system, such as capsules, powders, or liquid formulations, to maintain viability, stability, and efficacy during storage and transportation [141,142], and the final application of the produced probiotics in the intended fields, such as aquaculture, animal feed, and human health.

However, the current industrial production of marine probiotics is hindered by several factors, most importantly, the high cost of culture media required for optimal growth [28]. Optimal media for propagating marine probiotics should contain sufficient organic nitrogen, peptones, and protein hydrolysates from various sources. These media constituents are, however, expensive [28]. However, alternative sources of these essential elements/compounds from nature, such as organic marine wastes from fish and other life forms, are imperative. An example is the sourcing of fish peptones from different fish waste materials and by-products by Vázquez et al. [28] which resulted in 120 times better growth of the test probiotics *Pseudomonas fluorescens* and *Phaeobacter* sp. The development of these media nutrient requirements is ongoing through various studies and, with time, will ensure the scale-up of the production of proven probiotics for commercial use in the treatment of different medical conditions, including cancer and bacterial infections [143].

## 8. Limitations, Prospects, Future Perspectives

Studies in aquaculture have identified diverse marine bacterial species as potent probiotics [111,112,113]. Some identified bacteria can be considered safe (GRAS) for humans, while others are pathogenic. Since these marine probiotics have shown the potential to produce substances that could be of health benefit to humans, there is a need for innovative research to harness these benefits. In the development of marine probiotics, one major challenge is to isolate and identify potential strains [32,144]. In addressing low specificity and side effects associated with chemotherapeutic drugs and radiotherapy, there is a need to search for novel and non-toxic compounds from natural sources [79]. While the potential of terrestrial probiotics in cancer management has been explored, the function of marine probiotics is not yet fully understood. Several studies, including animal models and cell lines, have shown the promising therapeutic effects of marine probiotics. However, clinical trials are necessary to understand the therapeutic mechanisms [51] fully. Randomized, double-anonymized, placebo-controlled clinical trials are crucial to gaining broader acceptance in the medical community [49,57]. Although drug discovery and development from marine sources have inherent limitations, advances in analytical instrumentation, screening platforms, scalable synthetic approaches, and antibody-drug conjugates (ADCs) have expanded the clinical arsenal for therapeutic application [145]. In addition, omics approaches, including probiotic genome, metagenome, transcriptome, and metatranscriptome sequencing, are yet to be fully explored in cancer studies. These methods can enhance the detection and understanding of several bioactive mechanisms.

Biotechnological concepts and approaches are still evolving. In this review, we strongly recommend two possible biotechnological approaches that could yield beneficial products from these organisms; first, the selective identification and deletion of the virulence-causing gene(s) in the pathogenic marine probiotics while optimizing the expression of the genes responsible for producing the beneficial substances. The process affords the removal of the virulence factor and ensures the safety of use. This approach has yielded substances of pharmaceutical importance [146] and hindered the expression of patulin, a mycotoxin by *P. expansum* [147]. Second, the elucidation, manipulation, and optimization of the genes responsible for producing the bioactive agents using recombinant DNA (rDNA) technologies [148].

## 9. Conclusions

The need for alternative medicinal agents to synthetic/chemotherapeutic drugs and radiation (as it applies to cancer) in managing prevalent diseases is evident. Marine probiotics are, however, proven to be viable bioactive synthetic microorganisms. Specifically, *Pseudoalteromonas* spp., a group of marine probiotics, has shown potential therapeutic application against diseases, including cancer and bacterial-implicated diseases, through their activities and byproducts or metabolites. The production of therapeutic/bioactive agents from marine probiotics looks promising; however, the need for intensified research cannot be over-emphasized to enable optimal utilization of the potential therein.

## Data Availability

Data sharing not applicable.
